# Climate change and seismic resilience: Key considerations for Alaska’s infrastructure and built environment

**DOI:** 10.1371/journal.pone.0292320

**Published:** 2023-10-18

**Authors:** Matthew M. Turner, Majid Ghayoomi, Katharine Duderstadt, Jennifer Brewer, Alexander Kholodov

**Affiliations:** 1 Parsons Corporation, Boston, Massachusetts, United States of America; 2 Department of Civil and Environmental Engineering, University of New Hampshire, Durham, New Hampshire, United States of America; 3 Earth Systems Research Center, University of New Hampshire, Durham, New Hampshire, United States of America; 4 Department of Geography, University of New Hampshire, Durham, New Hampshire, United States of America; 5 Geophysical Institute, University of Alaska Fairbanks, Fairbanks, Alaska, United States of America; Mississippi State University, UNITED STATES

## Abstract

Alaska is one of the most seismically active regions of the world. Coincidentally, the state has also experienced dramatic impacts of climate change as it is warming at twice the rate of the rest of the United States. Through mechanisms such as permafrost thaw, water table fluctuation, and melting of sea ice and glaciers, climatic-driven changes to the natural and built-environment influence the seismic response of infrastructure systems. This paper discusses the challenges and needs posed by earthquake hazards and climate change to Alaska’s infrastructure and built environment, drawing on the contributions of researchers and decision-makers in interviews and a workshop. It outlines policy, mitigation, and adaptation areas meriting further attention to improve the seismic resilience of Alaska’s built environment from the perspectives of engineering and complementary coupled human-environmental systems.

## Introduction

**Fig 1:**
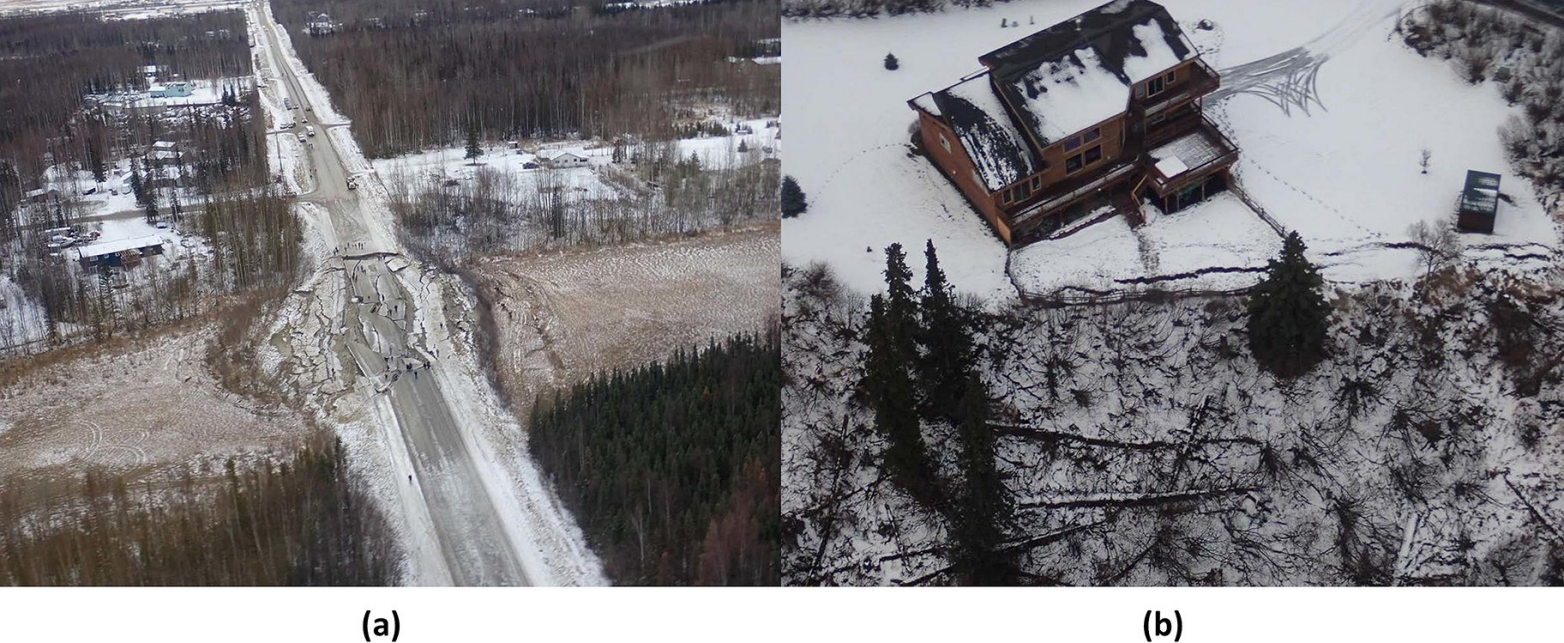
Damage caused by the 2018 Anchorage Alaska earthquake showing (a) lateral spreading along Vine Road close to Wasilla and (b) ground crack propagation along a sloping valley on the south side of Potter Creek in Anchorage.

The state of Alaska is highly vulnerable to the compound impacts of earthquakes and climate change. Since the 1960s, the direct financial cost of earthquakes and ensuing tsunamis in Alaska has far outweighed that of all other non-human-caused disasters combined [1]. Seismic impacts include coastal inundation, topographic rupture, mass movement of landslides and rockfalls, and soil liquefaction. Two of the most damaging earthquakes in 1964 (commonly referred to as the Great Alaskan Earthquake) and 2018 (commonly referred to as the Anchorage Earthquake) highlight the fragility of Alaska’s infrastructure, the former resulting in 115 deaths [2] (mainly from the ensuing tsunami) and about US$2.8 billion (in 2022 dollars) in estimated damages [3] and the latter causing significant damage to roads, railroad lines, and buildings [4, 5]. Examples of the damage caused by the 2018 Anchorage, Alaska Earthquake can be seen in Fig 1A and 1B.

Climate change is driving parallel processes such as permafrost thaw, water table fluctuations, and building subsidence that influence the seismic response of infrastructure systems. Alaska has experienced unprecedented levels of climatic warming, with statewide average temperatures increasing at a rate of about 0.4*°C* per decade since the 1970s, twice the global average [6]. Winter temperatures have increased by an average of about 3*°C* since the 1950s [6]. Arctic areas are experiencing more rapid warming than the state average [7]. By 2100, Representative Concentration Pathway scenarios [8] project annual average warming of at least 4–5.5*°C* in the state’s northern areas and 2–3.3*°C* in its southern areas [7, 9].

These changes are taking place in a state with a large geographic area, spatially dispersed population (.46 inhabitants per km^2^), and a limited road system. Alaska is ethnically diverse, with the highest Indigenous population among US states. The state is 60% non-Hispanic white, 15% Indigenous (with 20 recognized Native languages), and 25% Asian, Black, Pacific Islander, Latino, other, or multiracial. The cultural and livelihood differences among these groups are substantial, as they are between the majority of Alaskans who live in cities, towns, and suburbs, particularly along the coast, and smaller populations in rural areas accessible only by air or water. The Alaskan economy is highly reliant on oil and gas extraction, as well as government, military, fishing, other resource extractive industries, tourism, and subsistence hunting.

The challenges of seismic activity and climate change in Alaska are intricately connected, particularly in Arctic and sub-Arctic regions. The U.S. Global Change Research Program in the Fourth National Climate Assessment notes that as the Arctic region warms due to climate change, the permafrost layer that supports infrastructure such as buildings, roads, and pipelines may thaw, leading to soil instability and subsidence [7]. For example, Instanes and Mjureke [10] used the Arctic Climate Change Impact Assessment (ACIA) climate change models to project changes in soil bearing strength for seven sites throughout Alaska. Of the seven sites considered, Bethel Alaska experienced the largest reduction in soil strength (40%) between 1999 and 2090. It should be noted that Instanes and Mjureke [10] used an identical soil profile with the same thermal properties for all of the locations; therefore, their analysis can only be used as an indication of relative climate differences between locations. Nonetheless, soil strength deterioration can increase the risk of earthquake damage in some areas. Whitehouse et al. [11] found that climate change can also influence the magnitude of glacial isostatic adjustment in some regions. For instance, the melting of glaciers can lead to changes in the stress distribution along major faults throughout Alaska, potentially increasing the frequency and magnitude of earthquakes. Rollins et al. [12] discussed the impact of glacial melt on the 1958 moment magnitude, *M_w_*, 7.8 earthquake in Alaska, which triggered a landslide in Lituya Bay, resulting in a tsunami with a record-breaking maximum wave height of 1720 meters. Furthermore, the authors found that 23 of the 30 instrumentally constrained *M_w_*≥5.0 earthquakes in Southeast Alaska were promoted by glacial isostatic adjustment.

The impacts of climate change, such as permafrost thaw, melting of sea ice and glaciers, and water table fluctuations, can increase the vulnerability of infrastructure systems to seismic activity. This, in turn, may exacerbate the impacts of climate change by damaging critical infrastructure and further compromising the resilience of communities. Mitigating these risks requires a multidisciplinary and collaborative approach that accounts for the unique social, economic, and cultural contexts of Alaska. This qualitative study assesses and prioritizes concerns involved in earthquake resilience, amid a changing climate, at both the state and local levels in Alaska by leveraging informant interviews, an international workshop, and a review of the state of the art and identifies opportunities for enhancing capacity to prepare and respond at the community, academic, and governance levels.

In this study, resilience and vulnerability are viewed as closely connected concepts. Resilience refers to the ability of a system to withstand and recover from shocks and stresses [13–17], while vulnerability refers to the susceptibility of a system to damage or harm from such shocks and stresses [18, 19].

## Methods

**Fig 2:**
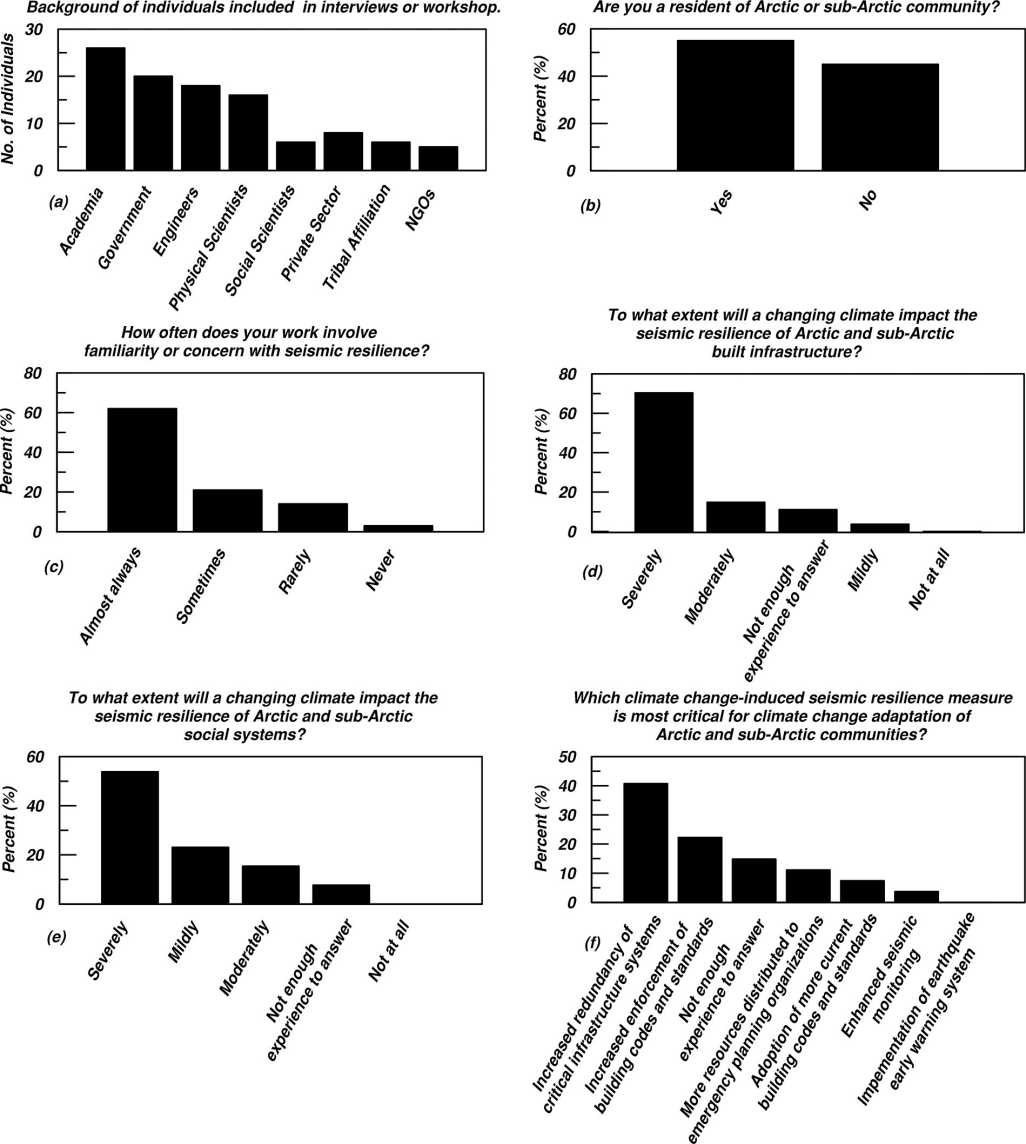
(a) Data summary showing the backgrounds of individuals included in interviews or the workshop. Note that several individuals fit more than one category and only professional tribal affiliations were documented, not ethnic identities. (b-f) Selected survey results of workshop participants.

To identify engineering opportunities to enhance seismic resilience amid a changing climate, an exploratory research effort synthesized several sources of information compiled in 2021 and 2022. These sources include a literature review, semi-structured key informant interviews, and contributions to a workshop titled “1^st^ International Workshop on Seismic Resilience of Arctic Infrastructure and Social Systems”. This workshop was hosted by the University of New Hampshire (UNH) and held in Anchorage, Alaska [20]. Ethics approval for this study was obtained from the Institutional Review Board of UNH (approval number 8498). The key informant sample was developed using chain-referral sampling to capture a range of expertise, mainly engineering, physical science, and government staff (such as emergency managers and planners), plus some representatives of Native communities (particularly Tribal government staff), social science, private sector, and a non-profit organization. Fifteen in-depth semi-structured interviews were conducted using a set of questions as an interview guide. These were performed via videoconference and subsequently transcribed, and thematically coded using qualitative data management software. Prior to the videoconference interviews, informed consent forms were provided to key informants through an electronically fillable PDF, subjects were requested to sign and return the PDF via email. An opportunity to ask questions about the consent and verbally confirm the consent was also provided at the start of the interview. Ten of the 50 participants in the 2.5-day workshop overlapped with the interview sample, generating a combined total of 55 interview and workshop participants (Fig 2A). Workshop participants were informed that workshop organizers may take notes and record audio and visual throughout the event. Participants were also informed that due to the relatively public nature of the workshop, participant anonymity may not necessarily be guaranteed. Workshops are typically based on a participatory method where attendees work together to develop ideas and form objectives related to a common issue or topic. This format provides opportunities for researchers to capture discussions, form topics, and identify priority needs of a community produced by participants who may not normally interact with one another outside of a facilitated discussion. In addition to formal presentations and informal discussions, 30 workshop participants completed a survey, and some survey results are highlighted in Fig 2B–2F. These survey questions helped generate a baseline understanding of attendees’ demographics, priorities, and concerns. About half of the workshop participants attended the workshop in-person in Anchorage, Alaska and the other half joined remotely via videoconference. The analysis and synthesis across these sources initially applied an engineering perspective, followed by a coupled human-environment systems perspective.

## Results and discussion

### Synthesis of engineering and physical science findings

**Fig 3:**
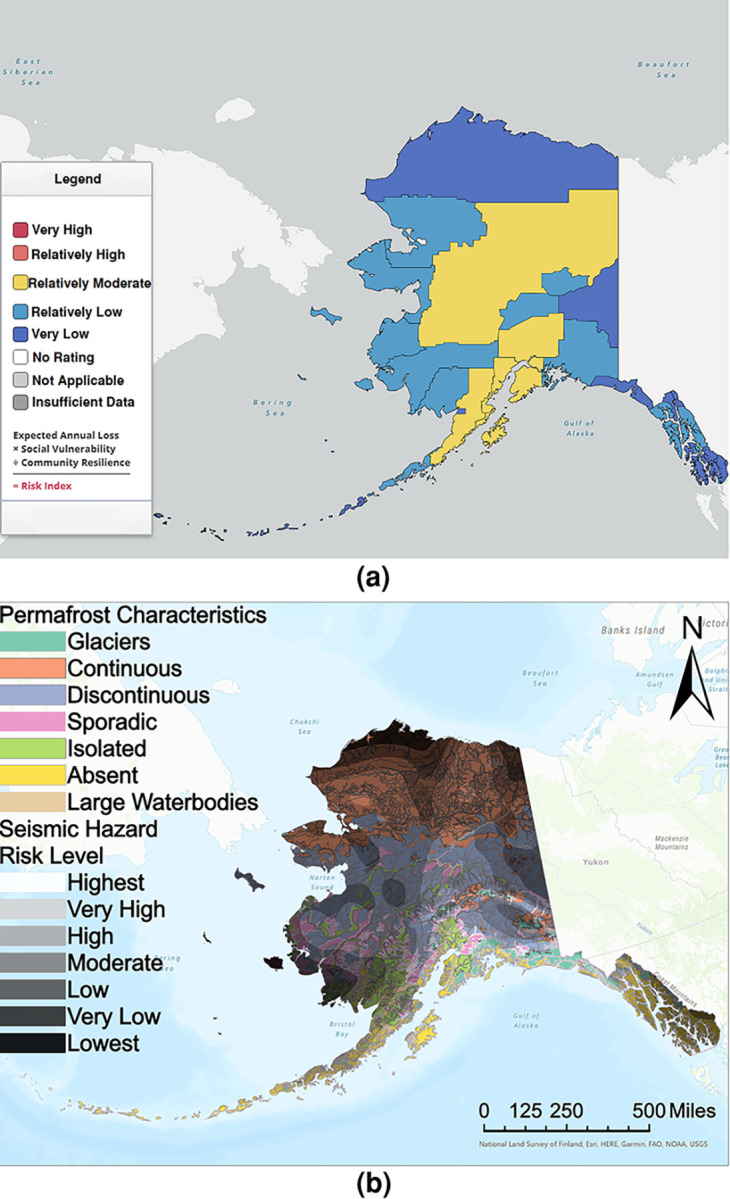
Maps showing (a) The National Risk Index variation in Alaska determined based on earthquake hazard [25]. Republished from [25] under a CC BY license, with permission from the Federal Emergency Management Agency, original copyright 2021 and (b) Comparison of the 2008 Permafrost Characteristics of Alaska [45] and the United States Geological Survey (USGS) 2018 Long-Term National Seismic Hazard Map (public domain) [46] overlaid on the USGS National Map (public domain). The 2008 Permafrost Characteristics of Alaska map is republished from [45] under a CC BY license, with permission from the Institute of Northern Engineering, original copyright 2008. Permafrost distribution is classified as continuous (>90% of the land area underlain by permafrost), discontinuous (50–90% of the area), sporadic (10–50% of the area), or isolated (<10% of the area) [47].

Hazards and vulnerability of infrastructure systems influence the risk of disasters. Past research addresses factors influencing vulnerability [21] and natural disaster losses [22–24]. Seismic risk and associated infrastructure vulnerability vary considerably across the state of Alaska. Fig 3A shows the National Risk Index (NRI) in Alaska determined based on earthquake hazard, combining earthquake expected annual loss, social vulnerability, and community resilience. Detailed documentation regarding the NRI and its derivation can be found in the NRI technical documentation [25]. Fig 3A indicates that Northern Arctic areas have “very low” seismic risk while central and south-central areas have “relatively low” to “relatively moderate” risk. The seismic hazard distribution and population density throughout Alaska greatly influence these variations. Climate change compounds these cumulative risks, threatening Alaskan communities with sea level rise, thawing permafrost, melting sea and land ice, and increased precipitation [7, 26–28]. Berman and Schmidt [29] estimated the economic costs of these climate-induced stressors in Alaska to be roughly $310 to $530 million (in 2022 US dollars) annually over the next 30–50 years. This estimate includes highly likely and easily quantified costs such as protection, maintenance, and repair of public infrastructure; community relocation; wildfire fighting and property loss, and replacement of ice roads. This estimate does not account for less certain or cascading direct and indirect costs such as those associated with private infrastructure, interruptions to drinking water or sewage services [30], temporary displacement of residents [31, 32], public health risks [33, 34], threats to monetized or subsistence livelihoods [34–37], and intensified labor demands to sustain formal and informal social networks [38]. It is important to note that the assessment of indirect losses is particularly important when evaluating the organizational and social aspects of climate change and earthquake damage on the entire community [39–41]. Coastal erosion resulting from climate-induced threats has caused several Alaskan communities to seek partial or total relocation [42]. Thawing permafrost is also generating widespread infrastructure damage to systems not at direct risk of erosion [7, 43, 44]. These damages shorten the useful life of buildings and lead to early retrofit and replacement.

Cognizant of these converging threats, workshop participants identified three leading climate-induced seismic infrastructure resilience concerns: (1) permafrost thaw and soil liquefaction, (2) lack of redundancy in lifeline infrastructure, and (3) cascading seismic effects. According to one structural engineer,

[Permafrost melt] may change how seismic waves travel if the soil is softer as it melts. But also, it starts creating problems for the piles [deep foundations] and they start losing capacity. You may end up with overturning failure… failure of a pile due to the fact that it doesn’t have the capacity you thought it was going to have.

Fig 3B synthesizes geographic information on seismic hazard [48] with permafrost distributions [45], uniquely identifying regions in Alaska where a changing climate will most likely affect seismic vulnerability and resilience. According to the figure, permafrost underlies about 75% of Alaska, roughly 800,000 km^2^, and is subject to the varied effects of climatic change [49]. Chadburn and coauthors [50] estimate that for every 1*°C* annual average temperature increase due to global climate warming, about 2.5 million square kilometers of permafrost in global arctic regions could be lost. In the discontinuous permafrost zone, where permafrost temperatures hover around the freezing point, the effects of rising temperatures are likely to be devastating. In these regions, even a small temperature increase may result in the total disappearance of permafrost [51]. Meanwhile, in the continuous permafrost zones, atmospheric temperature increase also raises the permafrost temperature, increasing the thickness of the active, seasonally thawed permafrost layer [52]. Estimates of the active layer thickness in Alaska from 2001 to 2015 suggest that this layer ranges from 0 cm in the North Slope to 300 cm in Coastal and Southern Alaska [53]. As a result of climate change, the thickness of the active layer is expected to increase by 20–30% across most of the permafrost area in the Northern Hemisphere throughout the 21^st^ century [54]. Fluctuations in active layer depth can influence other natural surface subsystems such as vegetation distribution and forest leaf cover [55, 56]. Overall changes in permafrost distributions will result in the discontinuous zone shifting further north, with reductions in the size of the continuous zone.

**Fig 4:**
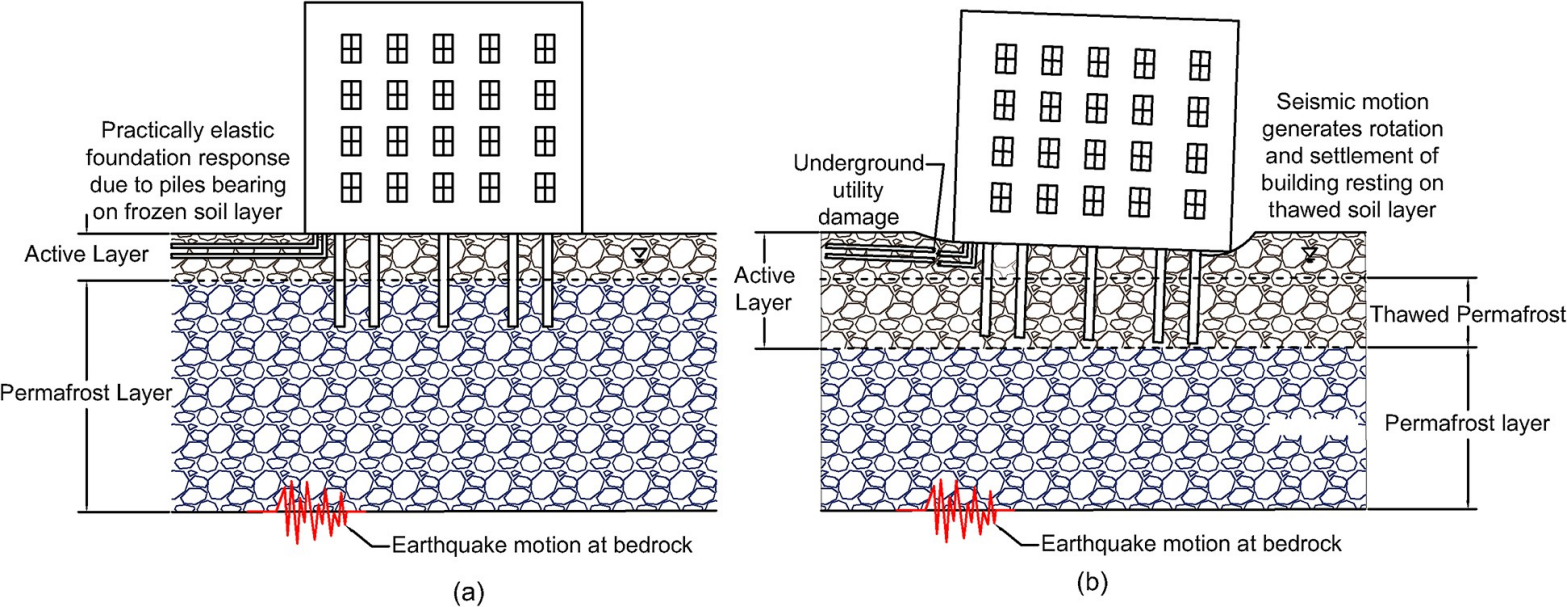
Conceptual impact of permafrost thaw on the seismic response of building-foundation systems showing (a) typical Arctic soil profile and (b) climate-induced permafrost thaw.

Changes in the mechanical properties of soils due to warming permafrost will alter the strength of soil layers. Most of the strength of frozen, fine-grained soil is generated by ice bonding, which creates a cohesive bond between soil particles [51]. Therefore, the extent of strength deterioration during thaw depends on the ice content of the permafrost. Permafrost with low ice content is generally considered thaw stable. When this permafrost melts, a layer of talik (perennially thawed soil) is generated between the upper, active layer, and the lower permanently frozen permafrost. In contrast, ice-rich permafrost is considered thaw unstable. As ice-rich permafrost warms, the presence of melted water reduces the strength and stiffness of these soils [10] leading to ground surface subsidence and thermokarst formation. Past research has shown that the seismic response of soil-foundation-structure systems is influenced by changes to the stiffness and strength of underlying soil layers [57–62]. Fig 4 shows the conceptual impact of permafrost thaw on the seismic response of a building, suggesting seismic motions propagating through once frozen soil will further deteriorate the soil strength due to changes in the stress and strain experienced by the material. Buildings and other structures resting on previously frozen but now saturated or near-saturated soil deposits that are subjected to intense ground motions may experience a sudden reduction in support. This loss in bearing capacity results in foundation settlements and rotations [61, 62], which in turn may damage underground utilities (Fig 4B). Therefore, it is prudent for practitioners to account for the variation and effect of permafrost thickness due to climate change over the design life of infrastructure systems (i.e., considering foundations depths and load transfer mechanisms).

Permafrost in the southern sections of Alaska has temperatures near the freezing point [51]. Soils in these locations are at the greatest risk of experiencing complete permafrost disappearance. According to Fig 3B, these regions also have the greatest seismic hazard, which means the potential risk of ground failure and infrastructure damage in southern Alaska will increase with a decrease in permafrost distribution. A practicing engineer noted,

That’s our geotechnical challenge in Alaska. It’s kind of the opposite of usual. When you build on frozen ground, you have to keep it frozen. Whereas normally you’re trying to put insulation down to keep it from freezing.

**Fig 5:**
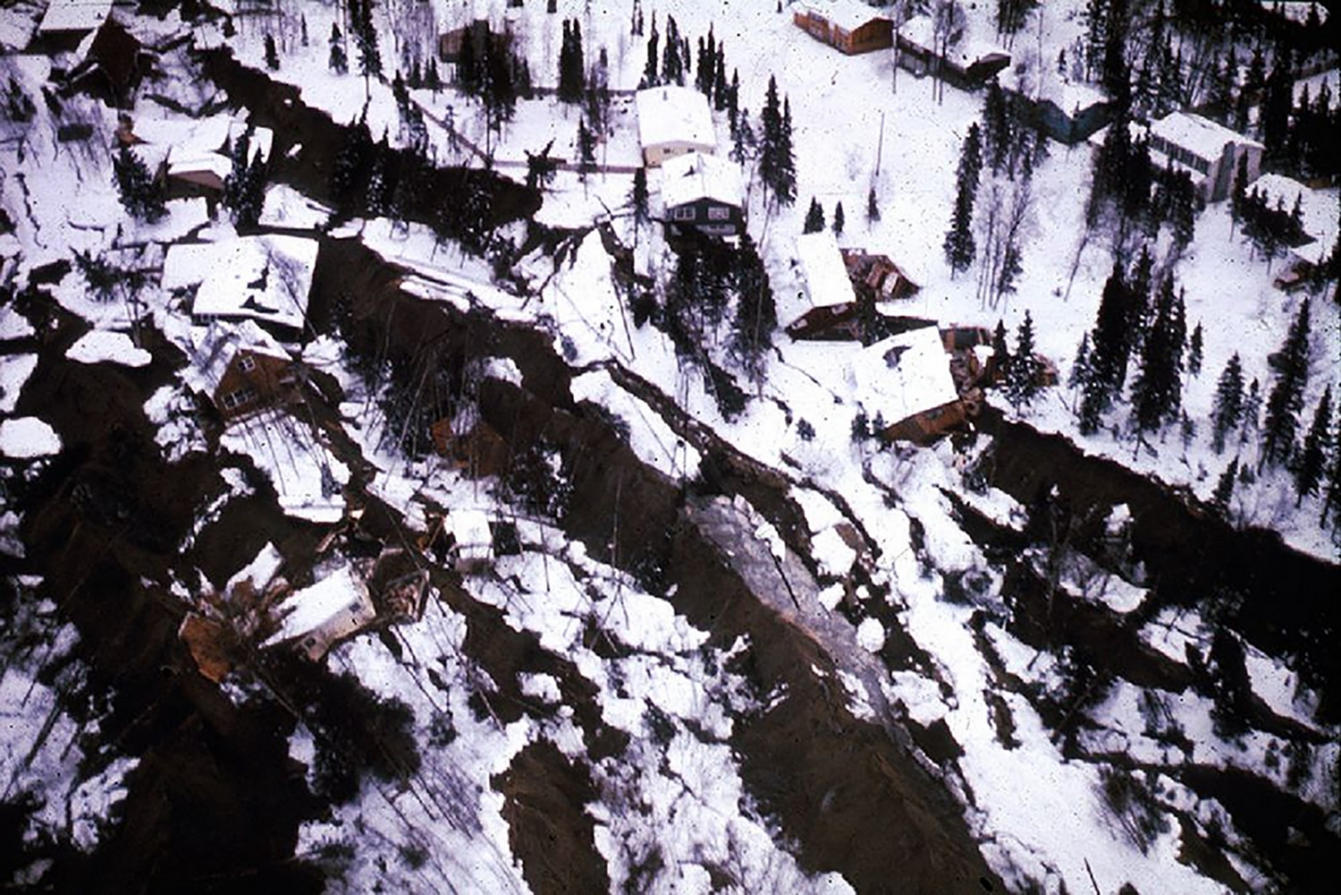
Liquefaction-induced long run-out landslide in Turnagain Heights Anchorage, Alaska generated by the 1964 Great Alaskan Earthquake.

Furthermore, permafrost degradation may increase the seismic liquefaction potential of soils [59]. During an earthquake, the contraction of saturated granular soil particles transfers stress from particle-particle contacts to pore water, leading to increased pore water pressure and a corresponding reduction in the strength of the material [63, 64]. Liquefaction occurs when the pore water pressure rises to a critical level and the behavior of the material changes from solid-like to liquid-like [65, 66]. After the earthquake, the dissipation of excess pore water pressure leads to ground settlement. In many cases, liquefaction-induced foundation settlements and rotations leave buildings and other infrastructure systems irreparable, even without above-ground structural damage [67]. Permafrost thaw can increase the risk of seismic liquefaction by altering the physical and mechanical properties of soil. As permafrost thaws, the water held in the ice-rich soil melts and occupies the pore spaces between the soil particles. This increased degree of saturation of non-frozen water changes the soil’s susceptibility to liquefaction during an earthquake. Consequently, the soil becomes more unstable and prone to soil settlements, landslides, and other forms of ground failure. Alaska has a history of well-known instances of liquefaction. An example of liquefaction-induced damage is shown in Fig 5, generated by the 1964 Great Alaskan earthquake, which led to a long runout landslide. This landslide destroyed 75 homes within the Turnagain Heights area of Anchorage [68]. A seismologist mentions,

We think at great length, in terms of strong ground motion during earthquakes. There’s a lot of [building] code that is devoted to making sure that structures can withstand strong shaking. That’s all worthless if the ground beneath the building moves, doesn’t matter. It doesn’t matter how you build it if the underlying soils are compromised.

### Building foundation technologies as potential solutions

Interview and workshop participants discussed several solutions and adaptation strategies to reduce the hazard of permafrost degradation on the seismic response of infrastructure systems. Generally, engineers seek to build infrastructure on ice-poor and ice-free terrain, where the amount of soil strength deterioration during thaw is minimal. For infrastructure systems already built on, or placed in, ice-rich permafrost, however, careful monitoring and evaluation of thaw-induced settlement and liquefaction potential of the foundation soil over the design life of the system can reduce risk. When building new infrastructure systems on ice-rich permafrost, foundation designs can compensate for permafrost thaw or actively/passively cool the underlying soil layers [69]. Active cooling relies on the use of systems with external power sources to maintain a certain permafrost temperature whereas passive approaches do not rely on external power.

**Fig 6:**
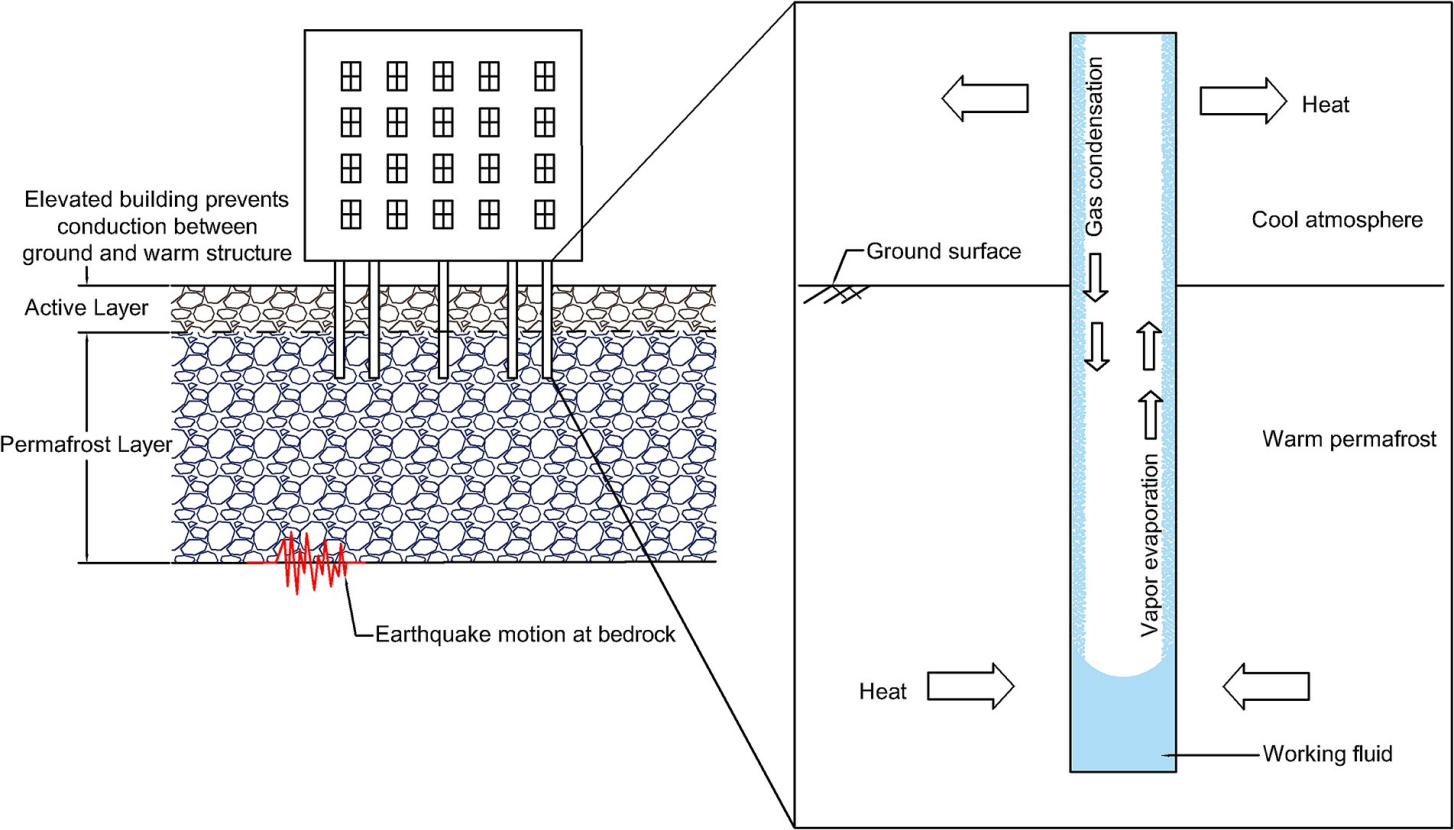
Engineering solutions to prevent permafrost thaw below a building [69]. This building features a crawl space between the building and foundation as well as several passive thermosyphons installed throughout the pile foundation system (commonly referred to as thermopiles).

Examples of passive systems can be seen in Fig 6 and include elevating a structure off the ground and using passive thermosyphons that employ a working fluid to convect thermal energy between the ground and atmosphere. Since the 1960s, thermosyphons have been used to stabilize foundations placed in continuous and discontinuous permafrost regions [70]. Thermosyphons have been incorporated into infrastructure design in more than 900 instances in Alaska [69], including about 120,000 thermosyphons installed along the Trans-Alaska Pipeline [71]. Notably, passive thermosyphons often only effectively function when the air is colder than the ground. Considering the current, and projected, climate change influence on the average winter temperature across Alaska, performance-based design would need to consider the reduction in the number of days in the year when passive thermosyphons function.

### Challenges of human-environment systems and building codes

From a coupled human-environment systems perspective, apparent engineering solutions encounter additional challenges. When asked about leading social concerns related to earthquake and climate impacts, workshop participants identified (1) capacities of small/local/Indigenous communities, (2) resource distribution, and (3) information access inequality. For example, access to crucial information related to seismic events and public safety is limited in remote areas, including Indigenous villages, though documents produced by the Alaska Earthquake Information Center and Alaska Division of Homeland Security and Emergency Management provide guidance to prepare for and recover from earthquakes at the household level. Participants suggested that opportunities exist to incorporate this information into in-person training-based modules.

Among possible options to increase seismic resilience, one advanced by several project participants was building code implementation to ensure the design of infrastructure systems that resist seismic loading. Widely used model US building codes (the International Building Code and International Residential Code) have included earthquake safety provisions since the 1990s but states and localities retain broad jurisdiction in the regulation of private construction and may adopt model codes fully, in part, or not at all. Construction in many smaller Alaskan communities falls under the jurisdiction of the state fire marshal, which requires building code adoption but not enforcement. A practicing engineer mentions,

Review of building plans, typically for larger structures in more remote communities, will undergo a fire safety review, making sure that the egress and all of these [similar] things are thought through. But there’s no structural engineer reviewing the structural calculations. There’s nobody looking into the real details.

In more populated areas, the local government may enforce codes, though requirements can vary between commercially built and owner-built homes, the latter being relatively common in Alaska. The value of building code implementation as earthquake protection was apparent in the 2018 Anchorage earthquake, which occurred after Alaska adopted the 2012 International Building Code. Within the Anchorage municipality, 40 buildings suffered significant structural damage. Of these, 38 were located in an area without code enforcement, suggesting that full code implementation can reduce property damage [72].

Practical challenges to building code enforcement in Alaska include difficulties of enforcement over large, sparsely populated areas, expensive and time-consuming transportation systems, and inclement weather. Furthermore, an interviewed geologist noted,

People don’t want to be told how to build their building. And people [in Alaska] still have that cowboy mindset of “I just want to build this how I want, and I don’t want the government to come in and tell me how it should be built.” But then they get in trouble when an earthquake happens and then they try to sell those houses to people that don’t realize that they’re not constructed to code and that’s dangerous…

In regions where less expensive housing exposes low-income communities to increased seismic hazards, the project participants proposed steps for building code implementation improvement including: (1) extending the code requirements to Anchorage surrounds; (2) requiring limited adoption of the residential building code for structures located in high-seismic zones elsewhere; and (3) requiring residential building inspectors be hired by the property owner and not the constructor/builder, to improve transparency and reduce conflicts of interest.

Other project participants raised related countervailing points, suggesting a cascade of questions and tradeoffs meriting further research exploration from a coupled human-environment systems perspective on adaptive capacity as a longer-term endeavor. Most regulatory change involves a reallocation of cost and benefits between public and private interests. These changes often result in differential impacts among heterogeneous social groups and entities. Significantly, social science and practitioner analyses of seismic and other environmental hazards find that reliance on centralized government and prevailing command-and-control models of resource mobilization can correlate negatively with resilience and that less tangible, social factors and capacity building can be significant [73–75]. One workshop speaker and advocate for Native communities urged consideration of alternative construction methods, avoiding technologies that are standard outside Alaska but may fail and cannot be easily repaired by local people with readily available materials. She pointed to reliance on bathroom plumbing as particularly problematic, whereas composting toilets or outhouses can be more reliable in remote, seismically active areas.

Social science studies of housing, construction, and regulatory oversight note that these activities are longitudinal processes that take place in complex and heterogeneous social contexts [73, 74, 76, 77]. In other words, they involve a wide array of social groups with diverse interests and knowledge bases, interacting in ways that vary over time and space. For example, building codes are often influenced by private sector interest groups with sufficient lobbying resources, are implemented per variable priorities of enforcement officers, and mainly affect new construction and renovations rather than existing construction [78]. In some housing markets, building codes can lower housing costs and associated transaction costs and/or raise property values through standardization and safety protections [79, 80]. In other markets, stringent or aspirational codes can shift the low-income housing market toward publicly-provided rental housing built for economies of scale, and away from local tradespeople who might build wealth in their neighborhoods through incremental, small-scale development, which tends to be more adaptive to social-environmental change [75, 79, 80]. Research supports the feelings of many independent builders and owner-builders that codes often advantage larger businesses with weaker social ties to tenants and business models focused on short-term profits rather than longer-term neighborhood development and quality of life [80].

Building code enforcement has differential effects on owner-occupied and rental properties and may reduce the availability of low-income housing in some markets [80, 81]. Codes can discourage the repurposing of older buildings and recycling of materials, stifle competition, and slow or speed certain kinds of innovation [79]. Some note that mandatory codes encourage building to the minimum standard, whereas training in best practices and voluntary or incentivized codes (such as streamlined permitting and inspections) can help establish self-replicating ethical norms while allowing for innovation [79]. Like any regulatory process, building codes can become mechanisms for discrimination or corruption [81]. Alaska is not immune from these phenomena as a resource-extractive frontier with relatively attenuated social networks, cultural tensions between Indigenous and settler populations, and limited news media coverage. Additionally, many communities are staunchly opposed to any increase in government regulatory oversight.

Helpfully, process models for the development of resilient housing and building codes that increase local capacity and are appropriate to local social and material contexts can be found in community and international development cases elsewhere [73, 77, 82]. Different construction approaches can have significant impacts on longer-term adaptive capacity, including local livelihoods, skill development, ecological knowledge, land tenure, cultural identity, and sense of place; norms of reciprocity, cooperation, and social aid; and sustainability of local resource use [73, 77, 83]. Community-based research methods could help articulate community values, identify resilience priorities, establish participatory and community-collaborative research methods, and identify and pursue shared goals [82, 84–86]. Indigenous and vernacular architectures and settlement patterns are often adapted to local environmental hazards and can sometimes be integrated with contemporary building techniques [73, 87, 88]. Related factors such as neighborhood density, architectural and landscape design, mixed-use development, and pedestrian and transit infrastructure also have powerful impacts on community resilience, such as by improving informal communications, mutual aid, upward economic mobility, and broad civic engagement [75]. Such decisions also have major climate change mitigation potential, without which public budgets will be depleted by escalating costs of environmental disaster.

## Conclusions

In this exploratory research, interviews and workshop contributors emphasized challenges posed by earthquake hazards and climate change for Alaska’s built environment, both from an engineering perspective and a coupled human-environment perspective. The array of concerns includes cumulative impacts of cascading seismic and climate events, such as destabilization of soils, sparse and diverse settlements spread across large distances, limited lifeline infrastructure and social networks, and variation in resource access and norms of decision making. Participants provided a clear and supported argument for the importance of considering permafrost thaw in seismic performance. Some proposed engineering solutions and mitigation strategies to address these risks and emphasized the potential role of building codes for public safety in relation to permafrost thaw and seismic performance. Others noted that building practices take place in complex human-environment decision contexts, which vary substantially over space and time, therefore meriting community-based approaches to identify policy gaps, opportunities, and limitations. This suggests that economic and social benefits can result from the consideration of permafrost thaw by engineers in relation to seismic performance of built infrastructure, including attention to building foundations and development of locally-appropriate building practices. Such actions are more likely to be effective and publicly supported if undertaken using methods that engage fully with a broad diversity of interests and knowledge bases, both in the framing of problems and their potential solutions.

## Supporting information

S1 File Inclusivity in global research. (DOCX)
